# Head and Neck Cancers in North-East Iran: A 25 year Survey

**Published:** 2017-05

**Authors:** Maryam Emadzadeh, Soodabeh Shahidsales, Amirhossein Mohammadian Bajgiran, Mahta Salehi, Toktam Massoudi, Zahra Nikfarjam, Maryam Salehi

**Affiliations:** 1 *Students Research Committee, Mashhad University of Medical Sciences, Mashhad, Iran*; 2 *Cancer Research Center, Mashhad University of Medical Sciences, Mashhad, Iran.*; 3 *Clinical Research Units, Mashhad University of Medical Sciences, Mashhad, Iran.*

**Keywords:** Demography, Epidemiology, Head and neck neoplasms, Iran

## Abstract

**Introduction:**

Cancers are among the worst noncommunicable diseases around the world. Head and neck cancers are ranked as the fifth most common cancers worldwide. As there are different distributions of risk factors around the world, the incidence of these cancers varies from one place to another.

**Materials and Methods:**

We conducted a descriptive analytic cross-sectional study, based on census-based records from the private oncology clinic in Mashhad, Iran. Data from 1,075 patients with head and neck cancers were analyzed from 1986 to 2010. We categorized the duration of study into five periods: 1986–1990, 1991–1995, 1996–2000, 2001–2005, and 2006–2010. Head and neck cancers refers to cancers originating from seven sites in the head and neck including the nasal cavity, oral cavity, pharynx, larynx, salivary glands, paranasal sinuses, and thyroid.

**Results:**

Data of 1,075 patients were analyzed. 66.2% were male. Mean ± standard deviation (SD) age at the time of diagnosis was 55.37±15.55 years. The most frequent type of head and neck cancer was larynx cancer (36%), followed by pharynx (28.5%), oral (17.5%), thyroid (6.8%), sinus (6.4%), salivary gland (4.10%), and nasal cancer (0.70%). although larynx cancer was the most frequent cancer over the whole study duration, there was a significant (P=0.04) difference in the relative frequency of these cancers across the five time periods. There was a significant difference in mean age between cancer categories (P<0.001). The only cancer with a different mean age at different time periods was pharynx cancer (P=0.02). There was a significant difference between sex and cancer categories (P<0.001).

**Conclusion:**

Laryngeal cancer was the most common head and neck cancer over the whole duration of this study. The differences in the patterns of other head and neck cancers could be due to geographical differences and also different risk factors and lifestyles all over the world. Further investigations in these fields are suggested in future studies.

## Introduction

Cancers are among the most serious noncommunicable diseases around the world, with 8.2 million deaths in 2012 worldwide. Sixty-five percent of these deaths and 48% of the 5-year cancer cases occurred in less-developed countries (1,2). Cancer is known as a leading cause of death in both developed and developing countries (3). In Iran, it is the third cause of death after heart diseases and accidents (4).

Head and neck cancers including oral cavity, nasal cavity, pharynx, larynx, paranasal sinuses, thyroid, and salivary glands ranked as the fifth most common cancers worldwide (5). These carcinomas account for more than 3% of all incident malignancies in the US (6). Oral cancer is the third most common cancer in developing countries (7), and has a higher burden in developing countries compared with developed countries (8,9). This cancer is among the most important cancers, with about 400,000 new cases and 130,000 deaths annually worldwide (10).

The economic costs and burden of disease could be reduced by wise and accurate planning (11). Improving knowledge about the epidemiology of different cancers has a key role in health care planning and can help policy makers to adapt their country`s health policy according to new research-based evidence (12,13).

There are geographic variations in incidence of head and neck cancers, reflecting different distributions in risk factors and also different lifestyles. Such risk factors include tobacco and alcohol use, radiation exposure, vitamin deficiencies, dietary habits, human papilloma virus (HPV) virus, occupational exposures and periodontal disease (14–19). The correlation between ethnicity and such cancers remains controversial due to contrasting results in different studies (20). In order to determine regional health priorities and identify the best services (such as preventive care), it is important to know the relative frequency, mean age at the time of diagnosis, and sex ratio of each type of cancer.

As people in different countries are exposed to different carcinogens and have different lifestyles and dietary habits, we decided to assess the relative frequency of head and neck cancers, determining the mean age at the time of diagnosis and the 25-year trend of head and neck cancers.

## Materials and Methods

In this descriptive analytic cross-sectional study, we conducted a census on registered data from the private oncology clinic in Mashhad, Iran. Mashhad is the second largest religious metropolis in the world and the second largest city in Iran (21). Mashhad is a referral city in terms of medical services in North-East Iran. Data on patients with head and neck cancers were collated from 1986 to 2010. Only records which were completely registered were included, and information on 1,075 patients were analyzed. We divided this duration into 5-year categories: before 1991, 1991–1995, 1996–2000, 2001–2005, and after 2006.

We divided head and neck cancers into seven major categories (in terms of original location): nasal cavity, oral cavity, pharynx, larynx, thyroid, salivary glands, and paranasal sinuses. The 25-year trend of these malignancies was assessed. In addition, we analyzed demographic information relating to age at the time of diagnosis, sex, job status and place of residence, including urban or rural. The pathology of each cancer was also described. Pathologic findings were placed into five groups: carcinoma, sarcoma, lymphoma, melanoma and not otherwise specified (NOS).

SPSS version 11.5 was used for all statistical analysis in this study. To evaluate the relationship between categorical variables, we used the Chi-square test. The normality of all data was assessed using the Kolmogorov–Smirnov test. Kruskal-Wallis and analysis of variance (ANOVA) were used for comparison between two groups or more in non-normal and normal distributions, respectively. A P-value <0.05 considered statistically significant.

## Results

**Fig 1:**
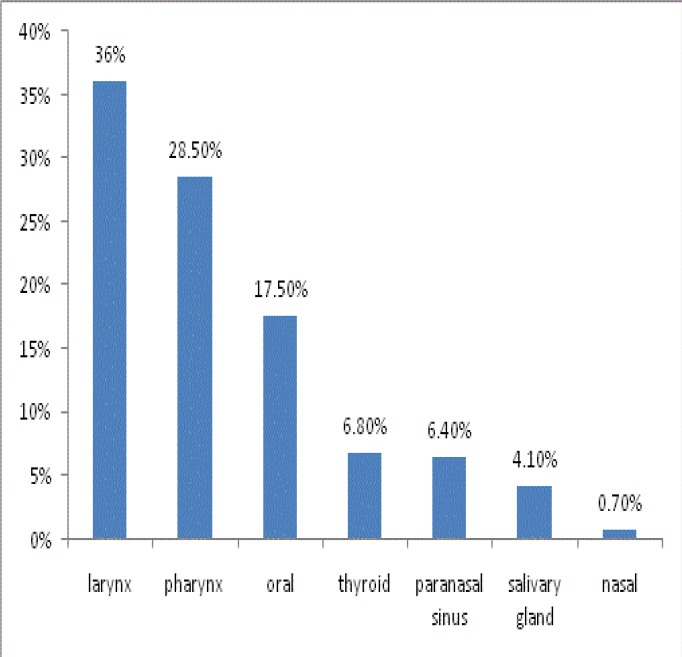
Frequency of head and neck cancers in study population

Data from 1,075 patients referring to the first private oncology center in Mashhad for head and neck cancers between 1986 and 2010 were assessed. In total, 66.2% of patients were male. The mean ± standard deviation (SD) age of patients was 55.4±15.5 years, with a range of 1–93 years. In total, 94.7% of patients lived in urban areas and 5.3% were rural. The frequencies of all seven cancer groups were assessed. We found that the most common head and neck cancer originated in the larynx (36%), followed by pharynx (28.5%), oral (17.5%), thyroid (6.8%), sinus (6.4%), salivary gland (4.10%) and nasal (0.70%) cancers, respectively (Fig. 1). Table 1 shows all seven branches with separate locations.

**Table 1:** Cancer categories divided by specific locations

**Cancer category (%)**	**Location**	**No. (%)**
Nasal (0.7%)	Nasal Cavity	4 (50%)
	NOS	4 (50%)
Oral (17.5%)	Lips (except skin)	10 (5.3%)
	Tongue	117 (62.2%)
	Gum	4 (2.1%)
	Mouth flora	17 (9%)
	Palate	13 (7%)
	NOS	27 (14.4%)
Larynx (36%)	Glotic	41 (10.8%)
	Supra and epiglotic	154 (40.6%)
	Subglot	10 (2.6%)
	NOS	174 (46%)
Pharynx (28.5%)	Tonsils	34 (11%)
	Oropharynx	5 (1.6%)
	Nasopharynx	146 (47.6%)
	Hypopharynx	107 (34.9%)
	NOS	15 (4.9%)
Paranasal sinus (6.4%)	Maxilla	55 (79.7%)
	Frontal	1 (1.5%)
	NOS	13 (18.8%)
Salivary gland (4.1%)	Parotid	31 (73.8%)
	Submandible	8 (19%)
	Sublingual	3 (7.2%)
Thyroid (6.8%)	Thyroid	73 (100%)

**Fig 2:**
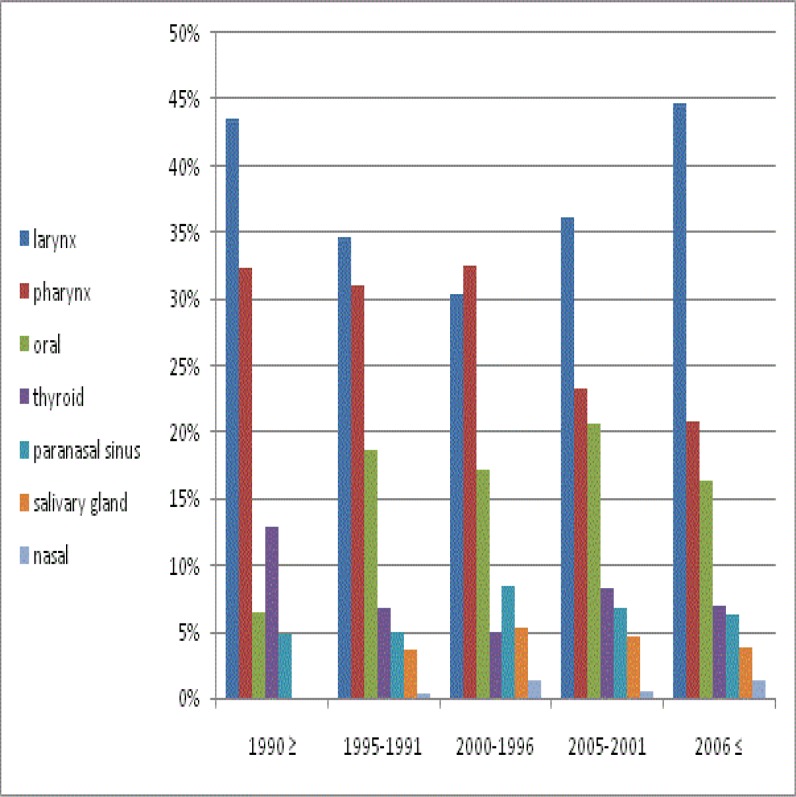
Trend of head and neck cancers according to five time periods

After dividing the study period into five intervals, we found that although larynx cancer was the most frequent cancer over the whole study duration, there was a significant (P=0.04) difference in the relative frequency of these cancers across the five time periods. Figure 2 shows the trend of head and neck cancers according to the five time periods.

The mean ±SD ages of men and women at the time of diagnosis were 56.4±15.04 and 53.4±16.3 years, respectively. 

There was significant difference between the mean age of men and women at time of diagnosis (P=0.007). Table 2 indicates mean age distribution at the time of diagnosis by sex in each cancer category. 

**Table 2:** Gender distribution and the mean age distribution by gender for each cancer

	Male (%)	Female (%)	Male/female ratio	Total mean age	Male mean age	Female mean age	p-value
Nasal	62.5	37.5	1.6	43.1±22.1	41.6±23.2	45.7±25	0.88**
Oral	49.5	50.5	0.97	59.8±15.9	58.4±17.2	61.1±14.6	0.33 **
Larynx	85.5	14.2	6.01	59.5±10.6	60±10.3	56.4±11.9	0.06 **
Pharynx	65.2	34.8	1.87	49.9±16.5	50.8±17.2	48.3±15	0.21*
Paranasal Sinus	51.5	48.5	1.06	51.5±20.4	55.1±19.1	47.9±21.4	0.15*
Salivary Gland	56.8	43.2	1.31	46.1±17.3	47.4±15.2	44.5±20	0.6*
Thyroid	30.1	69.9	0.43	55.1±14.9	57.3±16.4	54.2±14.3	0.37**

* Independent t-test

** Mann-Whitney-U test

As shown in this table, the lowest and the highest mean age occurred in patients with nasal and oral cancer, respectively (43.1±22.1 vs. 59.8±15 years). 

**Fig 3:**
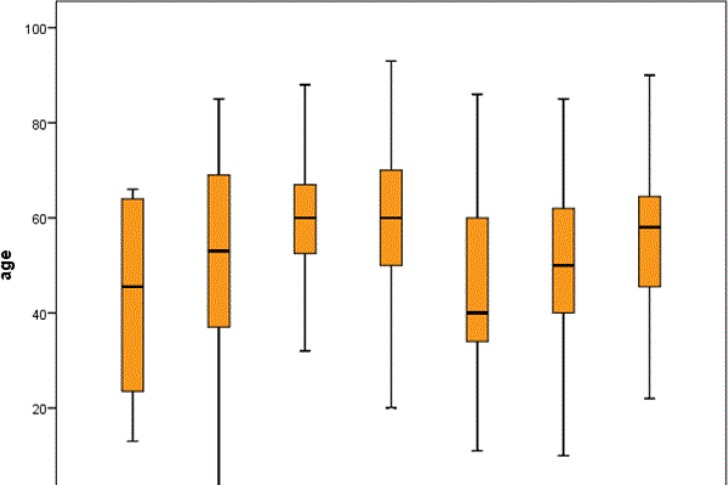
Box plot for age distribution in each cancer category.

There were significant differences in the mean age of diagnosis of patients among the different cancer categories, both overall and after separation by gender (P<0.001). A box plot for age in each cancer category is shown in Figure 3.

**Fig 4:**
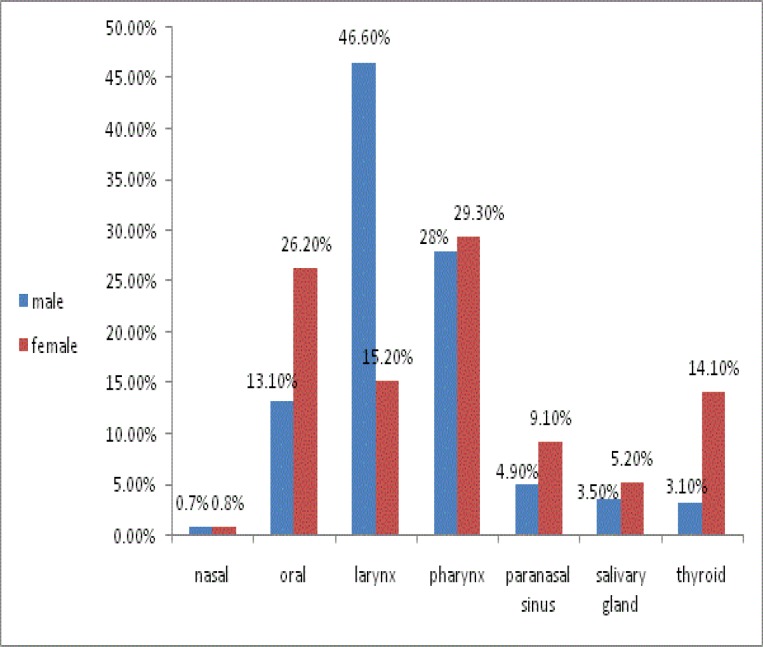
Cancer distribution according to gender

The distribution of head and neck cancers in male and female patients is shown in Figure 4.

As shown in this figure, larynx cancer is the most common cancer in males (46.6%), followed by pharynx (28%), oral (13.1%), sinus (4.9%), salivary gland (3.5%), thyroid (3.1%), and nasal cancer (0.7%). This order differs in females, with pharynx and oral cancers being the most frequent cancers. A Chi-square test indicated significant differences according to sex and cancer category (P<0.001). As shown in Table 2, oral and thyroid cancers are dominant in females, whereas others are more common in males. This test also demonstrates a statistically significant sex preference in larynx, pharynx, and thyroid cancer (P<0.001).

**Fig 5:**
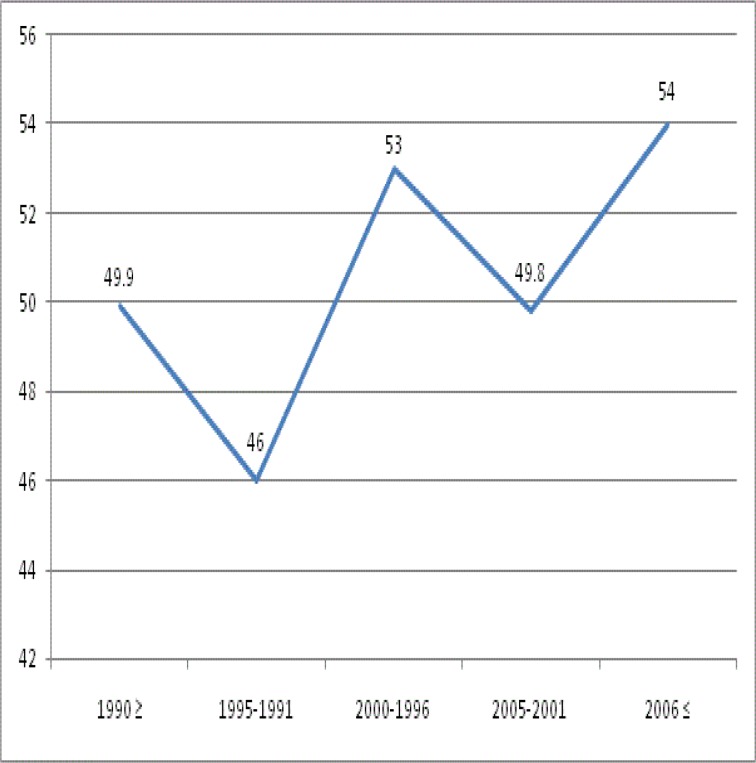
Trend of mean age in pharynx cancer

We analyzed the mean age at the time of diagnosis for each cancer in five time periods (Table.3). Only the mean age of patients with pharynx cancer differed significantly according to time period (P=0.02). Figure 5 shows the trend of mean age in pharynx cancer.

**Table 3:** Mean age of patients for each cancer type in 5-year interval.

**Cancer category**	**Time periods**					**p-value**
	**≤1990**	**1991–1995**	**1996–2000**	**2001–2005**	**≥2006**	
Nasal	-	34	52.75±23.86	13	43.5±19.09	0.36
Oral	51.25±16.19	57.77±15.6	60.56±15.33	61.21±16.85	62.27±17.26	0.36
Larynx	58.5±11.04	58.65±10.44	59.13±11.55	61.25±9.58	59.66±10.88	0.56
Pharynx	49.9±14.98	46.01±16.86	53.08±16.45	49.81±13.88	54±16.27	0.02
Paranasal sinus	55±13.22	49.89±21.91	48±20.15	51.33±19.92	65.4±14.49	0.22
Salivary gland	-	47.62±15.32	45±19.26	46.22±20.33	48.67±15.2	0.98
Thyroid	51.13±14.84	54.96±10.39	55.86±17.65	55.93±17.53	56.45±17.84	0.95

Assessment of the major pathologic groups shows that the most common pathology was carcinoma (93.5%), with lymphoma a distant second (4.8%). In addition, we found that in each cancer category, carcinoma had the highest frequency.

Squamous cell carcinoma (SCC) was the most common carcinoma (80.7%). After focusing on pathologic types of cancers in detail, we found that SCC has the highest frequency in pharynx, oral, and larynx carcinoma; whereas in nasal cancer, the frequency of SCC and olfactory neuroblastoma were equal (28.6% in each case). In salivary gland and thyroid cancers, the most common pathology was acinic cell carcinoma (ACC) (19.5%) and papillary thyroid carcinoma (50%).

## Discussion

In this study, the larynx was the most common site for head and neck cancers (36%), followed by the pharynx (28.5%). Nasal cancer had the lowest frequency (0.7%). In a previous study by Larizadeh in South-East Iran, the larynx was also the most frequent head and neck cancer (46.7%), followed by oral cancer (15.9%). In another study conducted in Rasht, Iran, oral SCC was the most common type of cancer in this region (22). In the study based on the national cancer registry in Iran, thyroid and larynx were the most common head and neck cancers, while salivary gland cancer was the least common. As data in the current study are derived from an oncologic center, and the most common treatment of thyroid malignancies is resection not chemoradiotherapy, thyroid cancer was not at the top of the list in our study (23,24).

Many studies have previously been reported with different results. For example, in two different studies in Brazil and Pakistan, cancers of the oral cavity were the most common head and neck cancers (25–27). In another study, Moore and colleagues found that oral cancer was the most prevalent neoplasm in Papua New Guinea and the nearby Solomon Islands (28).

In our study, larynx and pharynx cancers were the most common cancers in men, whereas pharynx and oral cancer were the most common head and neck cancers in women. This is in contrast with other studies reporting that nasopharynx cancer is more common in males, whereas females more commonly have oral cancer. In males, nasopharynx cancer is the fifth most common cancer (29). In one study conducted in Egypt, the highest incidence among males was for larynx and among females for gum and mouth cancers (30).

Although a U-shape trend of larynx cancer is seen in this study, a declining trend of this cancer has been seen in many studies, such as the study by Igissinov in Kazakhstan (31). In our study, we indicate an increasing and decreasing pattern of nasal and pharynx cancer. Davies and colleagues found similar results in the US (32). As shown in Figure 2, thyroid cancer decreased until 2000, and then showed an increasing pattern. Safavi and colleagues also found this increasing trend between 2004 and 2009 (22). We can also see an increasing pattern for oral and sinus cancers.

In our study, the mean age ± SD of participants was 55.4±15.5 years. This range was similar to that reported by Larizadeh in South-East Iran, of 53±17.2 years (23).In our study, the mean age at the time of diagnosis in each cancer type was similar to other studies; for example, in the current study the mean age of sinus and oral cancers was 51.5±20.4 and59.8±15.9, respectively, compared with approximately 54 and 58 in other studies (22).

In this study, oral and larynx cancers had the highest mean age at diagnosis (about 60 years), while the mean ages of paranasal sinus and pharynx cancers were about 10 years younger. In an epidemiological study conducted in Nepal, the mean age of the patients with laryngeal cancer was 60–69 (33). In an historical cohort conducted in Brazil, the mean age of patients with oral cancer was 44 years (34). Nasal cancer, with a mean age of 43, had the lowest mean age at the time of diagnosis. In the Poursadegh study, only 30% of patients with sinonasal cancers had a mean age under 45 years (35). In another study in Iran, the mean age of patients with nasopharynx cancer was about 47 years (36).

The mean age at diagnosis in our study was higher in men, except for oral and nasal cancers. The overall age standardized cancer incidence rate is almost 25% higher in men than in women (2). Overall, we can see that the mean age of patients with most cancers has increased during the study period. The mean age at the time of diagnosis for sinus cancer was very wide in five intervals of study duration (about 17 years). In the Safavi study, conducted in Iran, the mean age of patients with thyroid cancer was wide, the lowest mean age was in Ardebil province, but the highest was in West Azerbaijan (37).

In general malignancies are more common in men more than women (38,39). In this study, a female predominance was observed in oral and thyroid cancers, whereas all other cancers were predominant in males. These findings are similar to most studies in Iran. In the current study, the male/female ratio for larynx, pharynx, and thyroid cancer was 6.01, 1.87, and 0.43, respectively, compared with 7.6, 1.79, and 0.36 in the national cancer registry in Iran (24). Some articles confirmed our findings; for example in a retrospective study conducted in Kazakhstan, more than 90% of patients with laryngeal cancers were male (31). In another study, Choi found that thyroid cancer in men is declining (40). In other population surveyed, the incidence of nasopharyngeal cancer was higher in men. Our results were inconsistent with the results of other studies; for instance, in the Frydrych report, oral cancer was more common in men than in women (41,42). The study by Siddiqui in India indicated that the male:female ratio for head and neck malignancies was 3.1:1 (43). In total, 94.7% of patients who participated in the current study lived in an urban area. In the Attar study, urban residents were twice as common as rural dwellers (30). As with other studies, SCC was the most common carcinoma (6,44).

Since many articles emphasize tobacco as the most important known risk factor for the development of head and neck cancer, one of the limitations of this study was that we did not have access to smoking information for the participants. A lack of data relating to the patients’ body mass index and dietary habits were other limitations of this study, especially as it was recently reported that leanness is associated with an increased risk of head and neck cancer (45–52). As some of cancers may cured by surgery, these patients are not referred to radiotherapy centers, so data in this study do not cover all cancer patients. It should also be noted that this study was conducted in a private oncology center in Mashhad, suggesting that the patients may differ in some respects from typical cancer patients in terms of sociodemographic and risk factors. The other point to note is that in this study we included thyroid cancer in the category of head and neck cancers, while some classifications of head and neck cancer do not include thyroid cancer.

New studies report contrasting results on the association of ABO blood groups and head and neck cancers. For example, one study conducted in New Delhi in 2014 found that blood group A was a potential risk factor for the development of oral, esophageal and salivary gland cancers, whereas blood group B was found to be a potential risk factor for laryngeal cancer. Unfortunately, we did not have these data for the participants (53,54).

## Conclusion

According to our results, larynx cancer was the most common cancer across whole study duration, but there was little change over time in separated time periods. There was also some difference in the pattern of other head and neck cancers compared with some countries. This could be due to geographical differences and different risk factors and lifestyles all over the world. More epidemiologic studies and a greater focus on risk factors are suggested.
